# A Rare Case of an Intermittent Urinary Catheter Discovered Inside a Bladder

**DOI:** 10.7759/cureus.26736

**Published:** 2022-07-11

**Authors:** Konstantinos Pikramenos, Maria Zachou, Georgios Ntounas, Stamatios Katsimperis, Iraklis Mitsogiannis

**Affiliations:** 1 2nd Department of Urology, Sismanoglio General Hospital, National and Kapodistrian University of Athens, Athens, GRC; 2 Gastroenterology Department, National and Kapodistrian University of Athens, Athens, GRC; 3 Gastroenterology Department, Sismanoglio General Hospital, Athens, GRC; 4 Radiology Department, Sismanoglio General Hospital, Athens, GRC

**Keywords:** recurrent urinary tract infections, laser lithotripsy, intermittent catheter, urinary bladder stone, urinary lithiasis

## Abstract

Foreign objects inserted through the urethra, for sexual gratification and ending up in the urinary bladder, are rarely encountered. Patients usually present at emergency departments, reporting abdominal pain, recurrent urinary tract infections (UTIs), or haematuria. Only a few cases present without any symptoms and are incidental findings, commonly during diagnostic work-up for bladder lithiasis or recurrent UTIs. We report a case of an encrusted intermittent catheter, discovered in the bladder of a 72-year-old female patient, with a history of multiple sclerosis (MS) and recurrent UTIs. The foreign body was removed following laser defragmentation of the calculus. No indication of stone recurrence was documented during the six-month follow-up.

## Introduction

There are several reports of foreign objects within body cavities, inserted usually for sexual pleasure or forgotten during endoscopic manoeuvres [1-6]. Patients may be asymptomatic or present with lower urinary tract symptoms, which sometimes are attributed to a concomitant chronic disease and treated as such. When symptoms persist or recur, a detailed radiologic investigation to encompass all imaging modalities will usually reveal the true cause of the symptoms [7]. We present a case of a 72-year-old female patient suffering from multiple sclerosis (MS) and recurrent urinary tract infections (UTIs), who was diagnosed with a retained encrusted intermittent urinary catheter within her bladder.

## Case presentation

A 72-year-old female presented at the Accidents and Emergency (A&E) department reporting high fever (39.3°C) and mild haematuria for 24 hours. She had a known medical history of MS and dementia and reported several visits to the A&E department with various hospitalizations in the local hospital due to febrile UTIs, most commonly caused by Klebsiella pneumoniae and Candida albicans. The patient had been commenced on intermittent self catheterisation (ISC), during an episode of exacerbation of her neurological disease, two years prior. During her last visit to the local A&E department, three weeks ago, she had an indwelling catheter inserted. Noticeably, the patient had not been submitted to any radiographic or ultrasonic examination over the last 12 months, not even during her admissions, and was only treated symptomatically.

**Figure 1:**
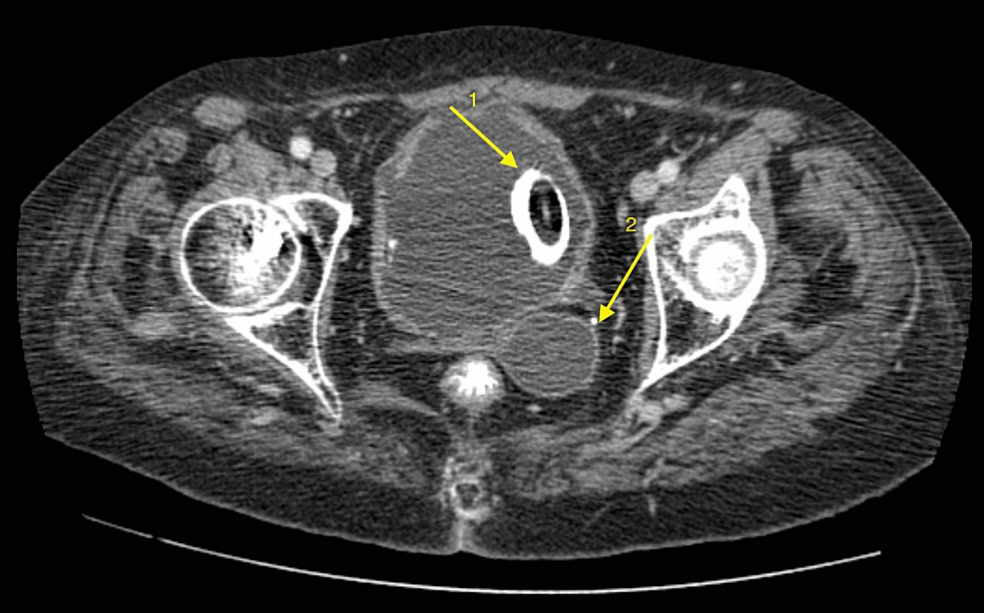
Computed Tomography of Bladder Foreign Body 1. Foreign body in urinary bladder encrusted; 2. Foley catheter in vagina

Laboratory tests revealed leucocytosis and elevated C-reactive protein (CRP) but normal urea and creatinine levels; whereas, ultrasonography reported a 2.5 cm stone within a thick-walled urinary bladder. A computed tomography (CT) scan showed a 2 x 2.5 cm bladder stone covering a foreign body; it also demonstrated the indwelling catheter to be located in the vagina (Figure 1).

**Figure 2:**
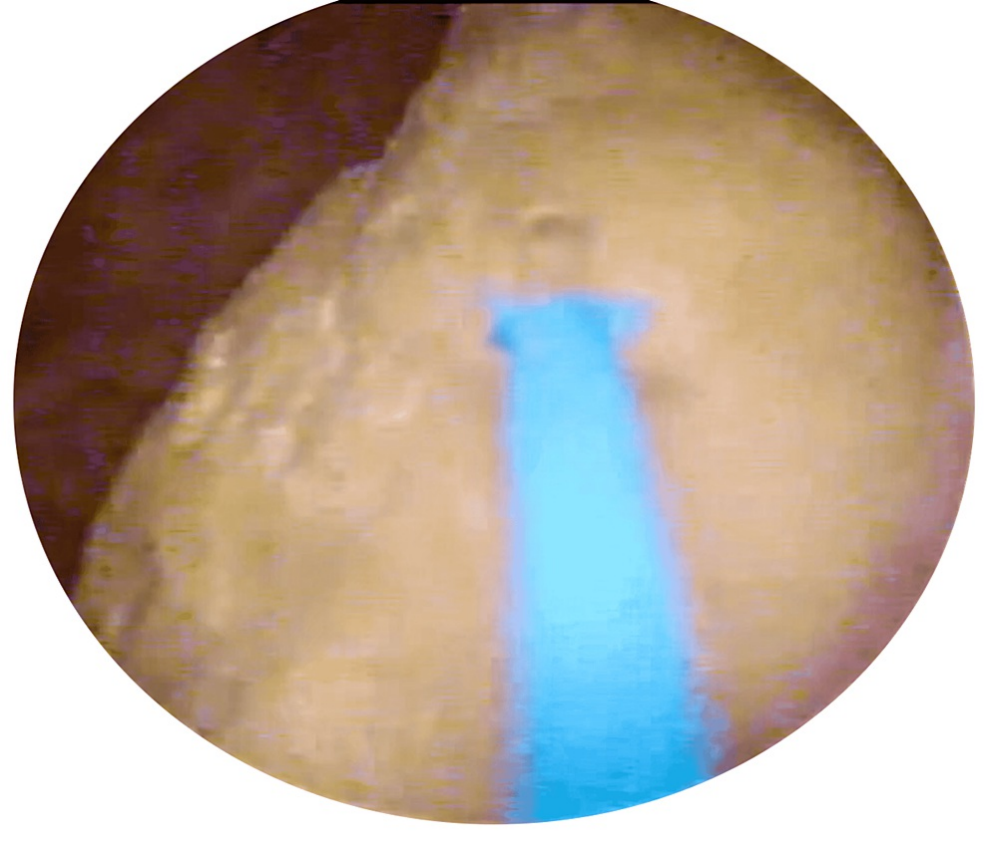
Laser Lithotripsy of Encrustation

**Figure 3:**
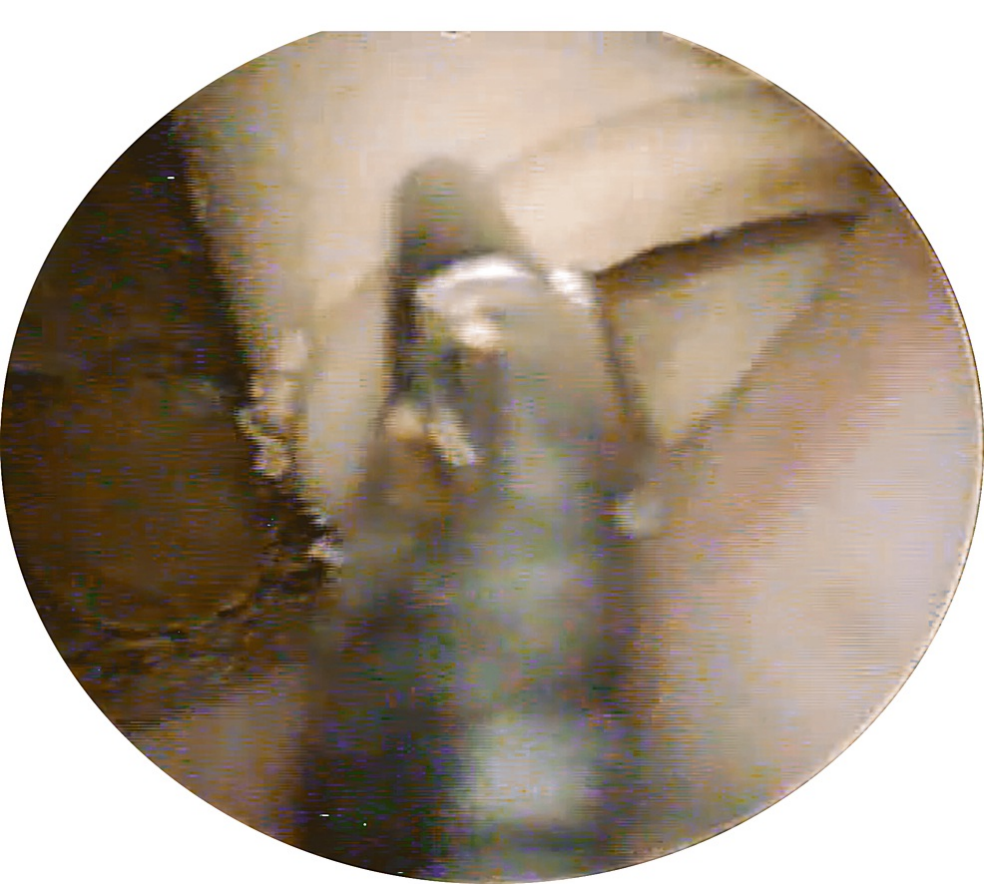
Endoscopic Removal of Catheter with Forceps

**Figure 4:**
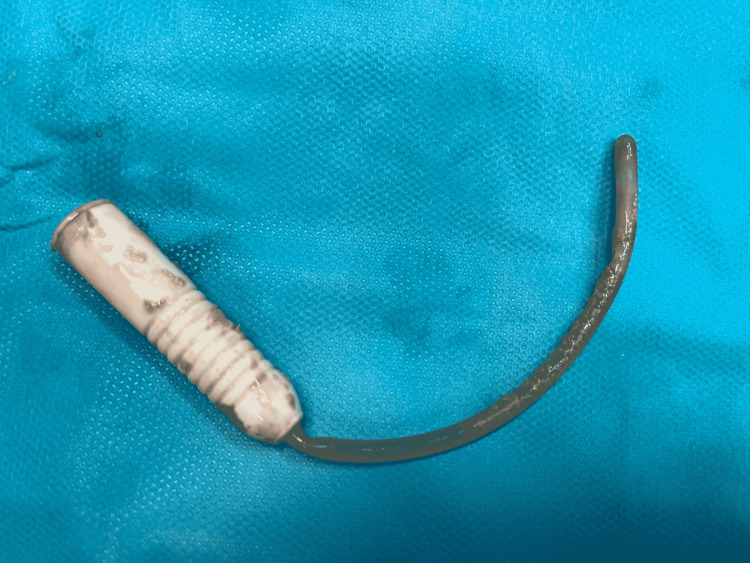
The Intermittent Catheter

The patient was admitted and commenced on broad-spectrum intravenous antibiotics. A cystoscopy was carried out, under spinal anaesthesia, which revealed an encrusted intermittent urinary catheter. Using a 365μm laser fibre, the encrustation was dusted and the catheter was removed with grasping forceps (Figures 2-4).

The patient became afebrile after two days and was discharged home on the 7th post-operative day. On follow-up two weeks later, a plain kidney ureter bladder (KUB) X-ray and an ultrasound scan were normal and the patient was instructed to visit the out-patient clinic periodically for radiology and ultrasonographic check. No recurrence of the stone was demonstrated at the six-month visit. 

## Discussion

Self-insertion of foreign objects into the genitourinary system is rarely reported and has been associated with autoeroticism, drug intoxication, or psychiatric disturbances. A wide variety of objects, i.e. iron wires, pins, needles, etc. have been documented [1-6]. Retained objects may cause lower urinary tract symptoms (LUTS), abdominal pain, haematuria, dysuria, and recurrent UTIs not responding to antibiotics. In some instances, patients delay seeking medical attention due to embarrassment. Diagnosis can be made by a plain KUB X-ray, ultrasonography, or a CT scan. Management depends on the size and shape of the retained object and is usually endoscopic, like in our case. Urine cultures are important for the administration of the proper antibiotic treatment, if required [1,7]. 

Retained foreign bodies may go unnoticed in the presence of concomitant chronic conditions causing similar symptomatology. In patients with MS, LUTS and/or frequent UTIs are not uncommon, hence a coexisting foreign object within the bladder may be overlooked. Our patient's symptoms, which were consistently monitored by local physicians, were attributed to her chronic disease, and a detailed diagnostic work-up could promptly unveil the problems that were neglected [8,9]. It should therefore be advocated that MS patients or those with chronic disease who experience persistent urinary tract symptoms should be assessed in full detail in order to rule out any other pathology as a causative factor of the symptomatology. 

Removal of the retained foreign objects is usually carried out endoscopically, although open surgery may be needed in some instances [1]. In our patient, the postoperative period was uneventful and in the six-month follow-up, no stone recurrence stone was observed.

## Conclusions

Foreign objects inserted into the urinary bladder are rare events, usually performed for sexual pleasure. The diagnosis may be delayed in the presence of a chronic disease-producing LUTS. Imaging is mandatory to accurately assess the location and size of the object, which in most cases can be removed endoscopically. Patients should be followed up periodically and psychiatric counselling should be advocated if required.
